# Pain-Induced Pessimism and Anhedonia: Evidence From a Novel Probability-Based Judgment Bias Test

**DOI:** 10.3389/fnbeh.2019.00054

**Published:** 2019-03-20

**Authors:** Benjamin Lecorps, Brent R. Ludwig, Marina A. G. von Keyserlingk, Daniel M. Weary

**Affiliations:** Animal Welfare Program, Faculty of Land and Food Systems, University of British Columbia, Vancouver, BC, Canada

**Keywords:** cognitive bias, emotion, dehorning, animal welfare, dairy cattle

## Abstract

Judgment bias tests (JBTs) use responses to ambiguous stimuli to infer emotional states in animals. However, with repeated testing, animals can learn to recognize the previously ambiguous stimuli rendering the test less effective. We describe a novel approach to this problem. Calves (*n* = 9) were trained in a spatial discrimination task to associate five locations with a specific probability of reward/punishment (Positive: 100%/0%; Near-Positive: 75%/25%; Middle: 50%/50%; Near-Negative: 25%/75%; Negative: 0%/100%). As predicted, calves showed increased latencies to touch locations that had higher probabilities of punishment and lower probabilities of reward. To validate our methodology for detecting mood changes, we followed calves in the hours after routine hot-iron disbudding, a time when animals were likely experiencing post-operative inflammatory pain. At 6 h after disbudding, when inflammatory pain was likely to peak, calves expressed increased approach latencies to the Positive, Near-Positive and Middle locations. These results suggest that calves perceived the value of the reward as being lower (i.e., anhedonia) or had lower expectations of positive outcomes (i.e., pessimism). When re-tested at 22 and 70 h after disbudding, we found no evidence of pessimism or anhedonia (i.e., latencies had returned to baseline). We conclude that our probability-based judgment bias task can detect pain-induced mood changes.

## Introduction

Judgment bias tests (JBTs) have been used to assess long-lasting emotional states (i.e., mood) in animals. In JBTs animals are trained to differentiate between cues that have positive and negative outcomes, and then are tested using ambiguous, intermediate cues; a decreased responsiveness to these intermediates (i.e., pessimistic judgment bias) is expected when animals are in a negative emotional state (Paul et al., 2005). However, repeated exposure to the intermediate cues can result in a loss of ambiguity as animals learn to associate these with a specific outcome (Roelofs et al., 2016). As ambiguous cues are commonly unrewarded, the loss of ambiguity may lead to decreased responsiveness (i.e., increased latencies to respond or decreased frequency of optimistic choices; Doyle et al., 2010; Barker et al., 2018), affecting the validity of the tests.

Several studies have attempted to prevent animals from learning to recognize ambiguous cues, for example, by using partial reinforcement for the training stimuli and thus rendering the lack of reinforcement for ambiguous cues less salient (Neave et al., 2013; Daros et al., 2014; Barker et al., 2016). In addition, the number of ambiguous cues presented can be minimized providing animals with fewer opportunities to learn (Hintze et al., 2018). In this study, we aimed to avoid the problem of declining ambiguity by intentionally training animals to recognize the different cues (in this case different locations) and associate these with specific probabilities of reward and punishment (e.g., from left to right: Positive: 100%/0%; Near-Positive: 75%/25%; Middle: 50%/50%; Near-Negative: 25%/75%; Negative: 0%/100%). Thus in our design, the task is not based on ambiguity but rather on reward probabilities that are known to the calves (even though the outcome for any specific trial is random within the constraints of that probability function). We predicted that calves would show higher approach latencies to cues associated with a lower probability of reward (and higher probability of punishment). To test the ability of this method to detect changes in mood we used hot-iron disbudding, a routine procedure known to cause postoperative inflammatory pain (Stafford and Mellor, 2011) and pessimistic judgment bias in calves (Neave et al., 2013). We predicted that animals would exhibit a pessimistic judgment bias (i.e., have a reduced expectation of reward and/or an increased expectation of punishment indicated by higher latencies to approach the intermediate locations) in the hours after disbudding, and that responses would return to baseline in the days following the procedure when pain had dissipated.

## Materials and Methods

### Animals

Nine female Holstein calves (BW: 38.3 ± 3.6 kg) were enrolled in the experiment from 10 to 35 days old. Within 6 h of birth, calves were separated from their dam and fed 4 L of >50 g/L IgG colostrum. Calves were housed singly (pen size 1.2 × 2.0 m) until 7 days of age after which they were moved to a double pen (2.4 × 2.0 m) and pair housed for the duration of the experiment. Calves were fed 4 L of whole pasteurized milk twice per day (at 08:00 and 16:00 h) using a nipple bottle, and had *ad libitum* access to water, hay, and grain. Fresh sawdust was added daily to the pens.

### Experimental Setup

**Figure 1:**
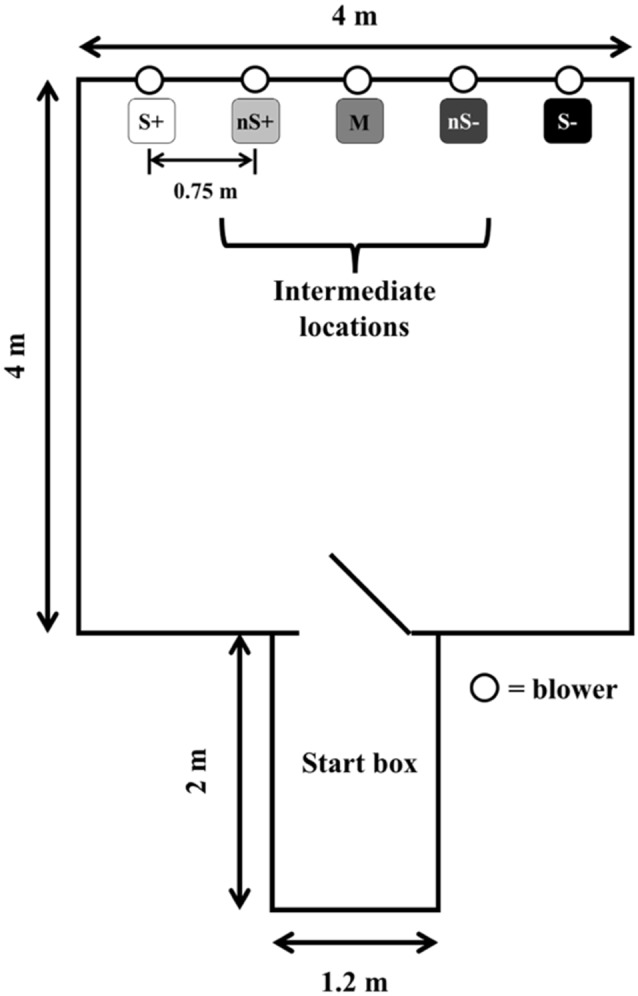
Experimental apparatus. All five locations were assigned a specific probability of reward/punishment (S+: 100%/0%; nS+: 75%/25%; M: 50%/50%; nS−: 25%/75%; S−: 0%/100%). Calves (*n* = 9) were trained for 20 days before being tested. The figure is adapted from Lecorps et al. (2018).

The experimental setting (Figure 1) consisted of the same apparatus described by Lecorps et al. (2018). The extreme right and left locations were designated as either the positive (S+) or negative (S−) locations (pseudo-randomly balanced across calves), and the intermediate three locations were designated as near positive: nS+, middle: M, near negative: nS−. Calves were familiarized with the apparatus in pairs for 10 min, 1 day before the training phase began.

### Training

Animals were trained individually in a go/no-go spatial judgment bias task to discriminate between five different locations each associated with a different probability of reward/punishment (S+: 100%/0%; nS+: 75%/25%; M: 50%/50%; nS−: 25%/75%; S−: 0%/100%). When calves were rewarded they were allowed to drink milk for 10 s. When calves were punished, they could not access milk from the bottle and instead received a puff of air to the face and had to wait 1 min before starting the next trial. For each trial, calves could choose to “go” (i.e., touch the bottle and receive the reward or punishment) or “no-go” (i.e., either wait for 30 s in the arena, or return to the start box to start a new trial). No-go responses were attributed the maximum latency. Training was divided into four phases (Table 1). Sequences used for training and testing as well as a video showing how calves responded to the different locations, can be found in the Supplementary Materials.

**Table 1:** Training and testing phases for the judgment bias task.

Phase	Locations					Description	Sessions	Cumulative trials
**Training**								
1	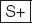					Training to associate positive location with milk reward	3	30
2	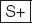				S-	Training to associate negative location with punishment	3	30 (15-15)
3a	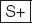	nS+			S-	Introducing the intermediate locations one at the time, in this case nS+	4	80 (24-32-24)
3b	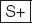		M		S-	Introducing the intermediate locations one at the time, in this case M	4	80 (24-32-24)
3c	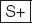			nS-	S-	Introducing the intermediate locations one at the time, in this case nS−	4	80 (24-32-24)
4	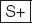	nS+		nS-	S-	This phase aimed at reinforcing the contrast between the two sides	2	40 (12-8-8-12)
**Baseline**				
1	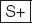	nS+	M	nS-	S-	All locations pseudo-randomly presented	1	20 (4-4-4-4-4)
2	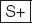	nS+	M	nS-	S-	All locations pseudo-randomly presented	1	20 (4-4-4-4-4)
**Tests**				
1 (6 h)	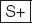	nS+	M	nS-	S-	All locations pseudo-randomly presented	1	20 (4-4-4-4-4)
2 (22 h)	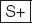	nS+	M	nS-	S-	All locations pseudo-randomly presented	1	20 (4-4-4-4-4)
3 (70 h)	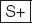	nS+	M	nS-	S-	All locations pseudo-randomly presented	1	20 (4-4-4-4-4)

Following training, baseline measures were recorded over two sessions each consisting of 20 consecutive trials. All probe locations were presented four times in pseudo-randomized sequences designed to minimize the number of consecutive rewards and punishments.

### Disbudding Procedure

Immediately before disbudding calves were sedated with a subcutaneous injection of xylazine (Rompun, 2%, Bayer Inc., Mississauga, ON, Canada; 0.1 mL/kg body weight; half-life 30 min), followed by a cornual nerve block on each horn bud (4 mL per side of 2% Lidocaine; Ayerst Veterinary Labs, ON, Canada; half-life 90 min). Five minutes later a hot-iron (Rhinehart X-30; Rhinehart Development Corp., Spencerville, IN, USA) was applied to each horn bud for approximately 15 s.

### Testing

Animals were tested at 6, 22, and 70 h after disbudding. Each test consisted of 20 consecutive trials in which probe locations were presented (four trials each) in pseudo-randomized sequences (using the same criteria as in baseline sessions).

### Statistical Analysis

Calves were allowed 30 s to approach the probe or return to the start box, but in 764 of 800 trials (i.e., 95.5%) they made a decision in less than 10 s (i.e., they either touched the bottle “go” or went back to the start box “no-go”). We therefore used 10 s as the maximum latency to avoid over-weighted outliers.

We used a curvilinear model including the latency to reach each location (as the response variable), and tested the effects of distance from the positive probe location. Calf was included as a random effect. Model residuals were scrutinized for outliers and normality. We then compared latencies to touch the different locations to the time calves took to go to the S+ location using non-parametric two-sample permutation tests because model residuals were not normally distributed even after logarithmic transformations.

We compared latencies between baseline and testing sessions at 6, 22, and 70 h after disbudding using three different models including the interaction between probe location (distance in meters with respect to S+) and session (two levels) with calf specified as a random effect. When the effect of session × location interaction was significant additional tests were performed by location.

## Results

**Figure 2:**
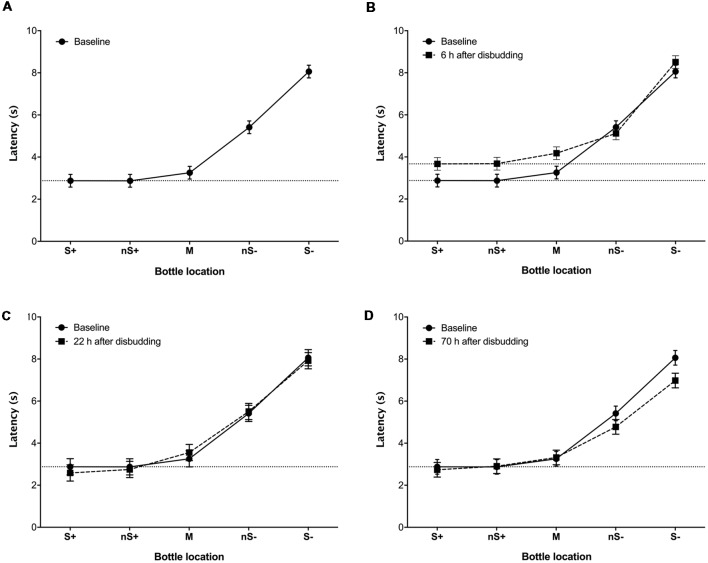
Least-square mean latency (±SE) of calves (*n* = 9) to reach locations associated with different probabilities of reward and punishment (S+: 100%/0%; nS+: 75%/25%; M: 50%/50%; nS−: 25%/75%; S−: 0%/100%) before **(A)**, and 6 **(B)**, 22 **(C)** and 70 h **(D)** after hot-iron disbudding. Each location was presented four times in a pseudo-randomized order (i.e., 20 trials). Baseline latencies to reach each location were calculated over two consecutive days of testing (40 trials; eight measures per location).

During baseline tests the latency to touch the bottle increased with the probability of punishment (χ^2^ = 525.4, *df* = 4, *P* < 0.0001; Figure 2A), indicating that calves were able to discriminate among the different locations. Calves showed longer latencies to approach the M (*Z* = 2.9, *P* = 0.001), nS− (*Z* = 6.9, *P* < 0.001) and S− (*Z* = 9.6, *P* < 0.001) locations compared to S+, but the latency to approach the nS+ cue did not differ from the S+. Calves tended to “go” (i.e., approach the test stimulus), regardless of location. For example, during the baseline sessions calves always showed a “go” response to the S+, nS+ and M locations, and almost always responded in this way to the nS− (91.7% “go”) but went less frequently to the S− (41.7% “go”).

At 6 h after disbudding, response latencies increased (*F*_(1,8)_ = 12.9, *P* = 0.007), with some evidence for a session × location interaction (*F*_(4,32)_ = 2.3, *P* = 0.08; Figure 2B). This interaction was driven by calves taking longer to touch the S+ (*F*_(1,8)_ = 25.3, *P* = 0.001), nS+ (*F*_(1,8)_ = 52.7, *P* < 0.001) and M (*F*_(1,8)_ = 21.9, *P* = 0.0016) after disbudding in comparison with the baseline. At 22 h after disbudding, no effect of testing session and no session × location interactions were observed (Figure 2C). At 70 h after disbudding response latencies were shorter than at baseline (*F*_(1,8)_ = 5.8, *P* = 0.043), with again some evidence of a session × location interaction (*F*_(4,32)_ = 2.0, *P* = 0.12; Figure 2D), driven by session differences at the S− location (*F*_(1,8)_ = 4.7, *P* = 0.061).

## Discussion

Our aim was to develop a method of assessing judgment biases that eliminates the risk that initially ambiguous intermediate cues lose ambiguity with repeated testing. Calves were trained to associate each location with a different probability of reward/punishment, such that the latency to approach these intermediate locations declined in relation to reward probability (and increased in relation to the likelihood of punishment).

Although the increase was clear for the nS− and S− locations, it was less clear for the most rewarded locations. Calves were statistically slower to touch the M location compared to the S+, but the time difference was small, and there was no difference in approach latency for the nS+ and S+ stimuli. Given that reward probability varied linearly across the five locations, one might expect that the responses should have also increased in a linear manner. However, according to the Expected Utility theory, decisions are based on how the reward and punishment are valued by the animal, and by the interaction between values and outcome probabilities (Loewenstein et al., 2008; Mendl et al., 2009). In this study, we controlled for outcome probability but not for the value of the reinforcers. Our finding of low latencies to touch the most rewarded locations may be explained by differences in the value of reinforcers, with the reward being more attractive than the punisher was aversive.

We intentionally varied rewards/punishers in relation to the generalization gradient to facilitate training, making it impossible to distinguish learned responses to intermediate reward probabilities from a stimulus generalization function. Curvilinear functions are common in JBTs, probably because the inherent value attributed to the reinforcers by the animals is usually unknown (e.g., Lyasere et al., 2017; Barnard et al., 2018). However, a previous study from our group used the same spatial learning task and the same reinforcers, but in this case calves were not trained to recognize the intermediate locations (Lecorps et al., 2018); in that study, the latency to approach the cues declined in a more linear manner in relation to proximity with the S+. The more curvilinear response in the current study suggests that the extensive training with the intermediates changed the animals’ perception of the reinforcers, most likely by decreasing the aversive nature of the punisher.

Although not systematically recorded, we noted different behaviors when calves approached the different locations. For instance, calves almost always only touched the nS− bottle with their nose, but directly latched onto the nipple of the nS+ bottle. The expression of micro-behaviors provides fertile ground for predictions based upon the different expectations calves had when approaching different locations; these predictions should be made explicit and tested in future research (Weary et al., 2017).

The current experiment was designed to detect changes in mood-based decision-making. After disbudding (when animals were likely in pain), calves responded more negatively (i.e., with longer approach latencies) to positive (S+) and the two closest intermediate locations (i.e., nS+ and M). This response biased was only found 6 h after disbudding when the inflammatory pain is thought to be most intense (Stafford and Mellor, 2011; Mintline et al., 2013).

Pessimistic responses are usually detected at intermediate locations, but we observed a bias that extended beyond the intermediate locations to include the S+ location. This response is consistent with some earlier work using traditional JBT (Harding et al., 2004; Novak et al., 2016). Reduced responding to the S+ may be driven by reduced motivation to access the reward (i.e., a decrease in the reward value), referred to as anhedonia and characterized by motivational and consummatory deficits in the consumption of specific resources (Treadway and Zald, 2011). Anhedonia is usually expressed when in a negative emotional state such as depression (Rizvi et al., 2016) or pain (Yalcin et al., 2014). Our results suggest that pain associated with disbudding induced mood changes that either triggered a loss in motivation (i.e., anhedonia) or lowered calves’ expectations of being rewarded (i.e., pessimism). Previous work in calves found a pessimistic bias to ambiguous probes after disbudding (especially to intermediate and near-negative cues; Neave et al., 2013; Daros et al., 2014), with no change in responding to the S+; this previous research used a different design (a color discrimination task) and a different response measure (go/no-go frequency) than that used in the current study. Latency measures are preferable when the number of observations is too low to accurately estimate the percentage of go responses, and some have argued that latency is a more sensitive indicator of motivation (Bateson and Nettle, 2015). Most importantly, the reduced responding to intermediate cues in previous work may have been due to calves learning that these cues were unrewarded, although this would not explain why the bias was focused at only one end of the generalization curve (Neave et al., 2013; Daros et al., 2014). It is important to note that previous work did not continue to test calves after the pain was expected to dissipate; in contrast, the current study shows that the negative bias disappeared when calves were re-tested at 22 and 70 h after disbudding. Whether hot-iron disbudding induces anhedonia needs to be confirmed. An alternate hypothesis is that calves were slower because the procedure affected their locomotion. In addition, as milk is the main component of the calves’ diet, the reduced motivation to go to most rewarded locations could be due to appetite loss (i.e., anorexia), another symptom of depression (Maes et al., 2012).

At 70 h after disbudding responses again differed from baseline. At this time calves showed a reduced latency to approach the S− location relative to baseline tests; this result can be interpreted as an optimistic response bias, perhaps associated with a positive contrast effect driven by calves no longer experiencing the inflammatory pain (Boissy et al., 2007). However, a positive contrast would be expected to cause a positive response bias also at other locations, including the nS− location. Calves were not tested between 22 and 70 h; this period between tests may have increased the animal’s interest in the task.

## Conclusion

We developed a probability-based judgment bias task for animals that reduces the risk that responses to intermediate cues are confounded with loss of ambiguity. Animals were trained to discriminate among locations associated with different probabilities of reward and punishment. Calves showed increased latencies to touch highly rewarded locations 6 h after disbudding, suggesting that they suffered from pain-induced pessimism and/or anhedonia. These results suggest that calves experience depression-like symptoms in the hours after disbudding.

## Data Availability

The dataset supporting this article has been uploaded as part of the electronic Supplementary Material.

## Ethics Statement

This study was carried out in accordance with the recommendations in the Canadian Council on Animal Care’s guidelines on the care and use of farm animals in research, teaching and testing. The protocol was approved by the Canadian Council on Animal Care (Protocol number: A16-0310).

## Author Contributions

All authors conceived and designed the study. BL and BRL carried out the experiment. BL conducted the analysis and wrote the manuscript. BL, BRL, DW and MvK revised and edited the manuscript. All authors approved the final version and agree to be held accountable for its content.

## Conflict of Interest Statement

The authors declare that the research was conducted in the absence of any commercial or financial relationships that could be construed as a potential conflict of interest.

## Supplementary Material

FIGURE S1 Sequences used for each of the four phases and the baseline/tests. During Phase 1, calves were trained to associate the positive probe location (S+) with the reward. During Phase 2, calves were trained to associate the negative probe location (S-) with the punishment. During Phase 3, calves were trained to associate each of the intermediate probe location (nS+, M, nS-) with a different probability of being rewarded/punished. During Phase 4, all probe locations except the Middle were presented to maximize the contrast between the positive and negative sides of the arena. All probe locations were presented during baseline measurements and judgment bias testing. The dot denotes punished trials for intermediate locations.

VIDEO S1 Calves performing the spatial learning task. Calves were trained to associate each location with a specific probability of reward/punishment (From Left to Right: Positive: 100%/0%; Near-Positive: 75%/25%; Middle: 50%/50%; Near-Negative: 25%/75%; Negative: 0%/100%). When rewarded animals were given 10s to drink milk. When punished, they had no access to milk, a blower was activated and calves had to wait 1min before the following trial.

DATASET Pain-induced anhedonia and pessimism: Evidence from a novel probability-based judgment bias test.
