# Supplementary material for: Pain-Induced Pessimism and Anhedonia: Evidence From a Novel Probability-Based Judgment Bias Test
Source: Front Behav Neurosci. 2019 Mar 20;13:54. doi: 10.3389/fnbeh.2019.00054 (PMC6435490; doi:10.3389/fnbeh.2019.00054)
Supplement: FIGURE S1 — Sequences used for each of the four phases and the baseline/tests. During Phase 1, calves were trained to associate the positive probe location (S+) with the reward. During Phase 2, calves were trained to associate the negative probe location (S-) with the punishment. During Phase 3, calves were trained to associate each of the intermediate probe location (nS+, M, nS-) with a different probability of being rewarded/punished. During Phase 4, all probe locations except the Middle were presented to maximize the contrast between the positive and negative sides of the arena. All probe locations were presented during baseline measurements and judgment bias testing. The dot denotes punished trials for intermediate locations. [file Table_1.DOCX]

Supplementary Material

# Supplementary Figures


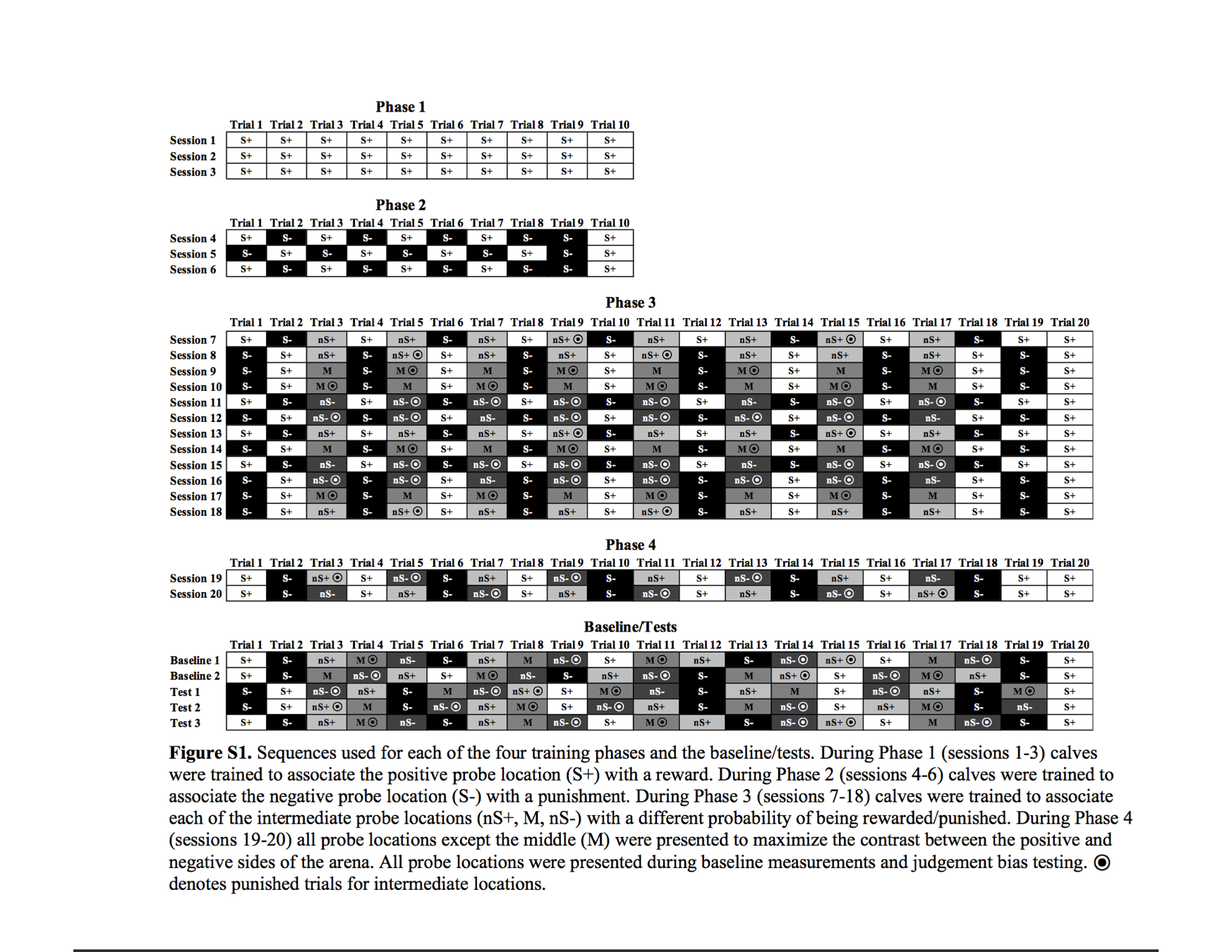


**Supplementary Figure 1.** Sequences used for each of the four phases and the baseline/tests. During Phase 1, calves were trained to associate the positive probe location (S+) with the reward. During Phase 2, calves were trained to associate the negative probe location (S-) with the punishment. During Phase 3, calves were trained to associate each of the intermediate probe location (nS+, M, nS-) with a different probability of being rewarded/punished. During Phase 4, all probe locations except the Middle were presented to maximize the contrast between the positive and negative sides of the arena. All probe locations were presented during baseline measurements and judgment bias testing. The dot denotes punished trials for intermediate locations.
